# Impact of gastrointestinal differences in veterinary species on the oral drug solubility, in vivo dissolution, and formulation of veterinary therapeutics ^#^

**DOI:** 10.5599/admet.1140

**Published:** 2022-02-14

**Authors:** Marilyn N. Martinez, Mark G. Papich, Raafat Fahmy

**Affiliations:** 1 Office of New Animal Drug Evaluation, Center for Veterinary Medicine, US Food and Drug Administration, Rockville, Maryland, marilyn.martinez@fda.hhs.gov; 2 College of Veterinary Medicine, North Carolina State University, Raleigh, North Carolina, mark_papich@ncsu.edu; 3 Office of New Animal Drug Evaluation, Center for Veterinary Medicine, US Food and Drug Administration, Rockville, Maryland, raafat.fahmy@fda.hhs.gov

**Keywords:** veterinary, gastrointestinal physiology, formulation considerations, solubility

## Abstract

Many gaps exist in our understanding of species differences in gastrointestinal (GI) fluid composition and the associated impact of food intake and dietary composition on in vivo drug solubilization. This information gap can lead to uncertainties with regard to how best to formulate pharmaceuticals for veterinary use or the in vitro test conditions that will be most predictive of species-specific in vivo oral product performance. To address these challenges, this overview explores species-specific factors that can influence oral drug solubility and the formulation approaches that can be employed to overcome solubility-associated bioavailability difficulties. These discussions are framed around some of the basic principles associated with drug solubilization, reported species differences in GI fluid composition, types of oral dosage forms typically given for the various animal species, and the effect of prandial state in dogs and cats. This basic information is integrated into a question-and-answer section that addresses some of the formulation issues that can arise in the development of veterinary medicinals.

## Introduction

Drug thermodynamic solubility (*C*_S_) is one of the determinants of drug oral bioavailability. The US Pharmacopoeia (USP) general chapter, GC<1236> [1], defines *C*_S_ as “the maximum quantity of a substance that can be completely dissolved at a given temperature, pressure, and solvent pH”. It is only upon in vivo solubilization that a drug can penetrate biological membranes. Once traversing the enterocyte membrane, it is either effluxed back into the intestine, metabolized within the enterocyte, or transported into the portal (or lymphatic) circulation [2,3].

The conditions influencing in vivo drug solubility can differ markedly across an individual’s gastrointestinal (GI) tract, between individuals of a given species or between species. Unfortunately, as compared to humans, there remain many gaps in our understanding both of species-specific differences in GI fluid composition and the impact of food intake and dietary composition on in vivo drug solubilization. Furthermore, with the exception of cattle, there is little to no published information on GI fluid composition as a function of animal age, breed, or diet (see details below). These gaps can lead to uncertainties with regard to how best to formulate pharmaceuticals for veterinary use or the in vitro test conditions most predictive of in vivo product performance.

This overview describes species-specific factors that can influence in vivo drug solubility and potential formulation approaches to overcome bioavailability challenges. Discussions focus on the “major” target animal species within the US (dogs, cats, horses, pigs, swine, cattle and chickens and turkeys) www.farad.org/us-food-animals.html.

## General considerations

The first step in understanding how species differences in GI fluid composition can influence in vivo product performance and drug solubility is to appreciate the difference between dissolution rate and substrate solubility (*C*_S_). In that regard, variables influencing dissolution rate can be described by the Nernst-Brunner equation:

(1)

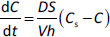


where *C* is the solubilized concentration at time *t*, *C*_S_ is the solubility of the substance in question, *S* is the surface area of the dissolving particle, *D* is the diffusion coefficient, *h* is the thickness of the diffusion layer, and *V* is the volume of the diffusing medium [4]. With regard to *h* and *D*, the species differences in fluid viscosity need to be considered. *V* can also vary as a function of species and prandial state.

To appreciate how GI fluid composition may influence in vivo values for *C*_S_, it is necessary to recognize the factors that can influence the interaction between a drug, its salt form, drug solid-state characteristics, and the aqueous environment within which the drug molecule will dissolve [5]. In vivo factors to consider include:

- Fluid viscosity [6]. As discussed later, this can be a particularly important factor affecting oral drug bioavailability in dogs and cats when administered in the fed versus fasted state.
- Solubility-enhancing surfactants (including those incorporated into the formulation and natural surfactants [7-9]. In this regard, the differences in bile salt composition may influence the nature of drug solubilization across the various veterinary species (an important gap remaining in our understanding of physiological differences as a function of species, diet and breeds).
- Fluid volume versus dose. Within veterinary medicine, formulations are typically approved for administration on the basis of an animal’s body weight (BW). However, BW and gastric fluid volume may not scale directly. This issue can be particularly problematic in dogs where although the body size can range from small miniature breeds to giant breeds (a range that can span 4 to 230 Lbs), residual gastric fluid volume is typically limited to the small amounts of water consumed. Estimates of gastric fluid volumes in food-producing animal species and horses as provided in CVM’s GFI #171 [10] are included in Table 1.

## Oral dosage forms

The development of species-specific formulations necessitates adapting dosage forms to species-specific constraints in drug delivery, whether attributable to palatability issues, husbandry practices or pet owner compliance. Examples of veterinary dosage forms for the various species have been described in the Merck Veterinary Manual [11] and are listed in drug formularies and handbooks [12].

Dogs and cats: Tablets, capsules, solutions, and suspensions. Given the upsurge of interest in chewable dosage forms, this topic is further discussed in detail later in this review.
Horses: Solutions, suspensions (typically administered by nasogastric tubes), pastes (applied to the tongue), syrups, and granules (as medicated feed).
Cattle: Medicated feed, drinking water, and oral boluses.
Pigs: Medicated feed and oral solutions.
Poultry: Medicated feed and drinking water.

## Unique dosage considerations in veterinary medicine

### Administered dose

In contrast to human medicine where the dose is titrated to effect, veterinary pharmaceuticals are typically administered on a mg/kg basis. An exception is the cat, where oral formulations are typically designed to be administered as either one tablet or capsule per cat or as liquid formulations that deliver a specific fluid volume (e.g., 1 mL) per cat.

In both the mg/kg and unit/cat dosing paradigms, the importance of recognizing the relationship between gastric fluid volume and BW is underscored. In veterinary species, the fasted gastric fluid volume reflects residual liquid plus ad libitum water consumption. In contrast, the human gastric volume is assumed to reflect the consumption of 8 ounces (240 mL) of water. Because the Biopharmaceutics Classification System (BCS) drug solubility assessments (typically expressed as a “dose number” (*D*_o_)) are based on the highest approved human oral dose in 240 (or 250) mL of fluid, where *D*_O_ = (dose strength/240mL)/drug solubility, we cannot simply extrapolate human BCS drug solubility classifications to non-human species (e.g., Papich and Martinez, 2015) [13].

Another challenge confounding veterinary therapeutics is that upon occasion, the same medication (same formulation) may be administered to cats and dogs. However, the substantial anatomic and physiologic differences between these two species can result in differences in drug solubility and the fraction of administered dose absorbed. Examples of how this may influence drug oral drug solubility and absorption are discussed later in this review.

### Canine-specific BCS challenges

Given the wide range of canine body weights (approximately 4 lbs for a Chihuahua or Pomeranian to 230 lbs for a male English Mastiff), the question is whether *D*_O_ needs to be determined on a breed-specific basis. The percent contribution of the GI tract to total BW can differ as a function of dog breed. For example, considering the miniature Poodle versus the Great Dane, the GI tract comprises 3-4 % of the total BW of large breeds but comprises 6-7 % BW in smaller breeds [14]. Moreover, breed differences in intestinal length may affect the relationship between the rate of in vivo dissolution and the concentration of solubilized drug driving absorption across the intestinal segments. We note that a significant positive correlation was observed between BW and fecal water content in 60-week-old dogs [15], raising the question of whether there may, in fact, be some difference in the fraction of drug dissolved within the colon of large versus small breed dogs (especially if rapid intestinal absorption is not available to maintain in vivo sink conditions).

While puppies have been observed to exhibit a longer orocecal transit time (OCTT) than adults, this difference was only statistically significant in large canine breeds [16]. Body size did not significantly influence the OCTT across breeds of adult dogs. In that study, OCTT was defined as the interval from ingestion of the meal to the time at which a marker compound was detectable in plasma. Thus, it primarily reflected the duration of gastric emptying and small intestinal transit. In contrast, when measuring mean total gastrointestinal transit time (TTT, time from consumption to defecation), Hernot et al. (2005, 2006) observed a positive correlation to canine BW [16,17]. This was attributable to size-associated increases in large intestinal transit time. Assuming that most drug absorption occurs in the small intestine, it is likely that canine differences in BW, TTT, and fecal quality will not lead to drug solubility-associated differences in oral bioavailability (with the exception of situation involving colonic absorption as noted above).

### Fed versus fasted state

Similar to humans, the canine and feline GI tracts can exist either in a fed or fasted state. While we know that canine intestinal transit times tend not to be influenced by the presence or absence of food [18,19], to date, there is an absence of published information on the postprandial GI fluid composition of dogs or cats.

When evaluating human drug products, the US FDA recommends that formulation effects be considered under both fed and fasted conditions because, in some cases, food can magnify formulation-associated differences in product bioavailability [20]. However, due to constraints encountered in veterinary medicine, in vivo bioequivalence studies are typically conducted in fasted dogs and cats (VICH GL52) [21]. Exceptions are those drug products specifically labeled for administration in the fed state. Furthermore, certain oral dosage forms for dogs and cats are formulated as chewy treats or are administered in treat-like pouches, which itself is likely to induce a fed or semi-fed state upon ingestion.

**Figure 1.**
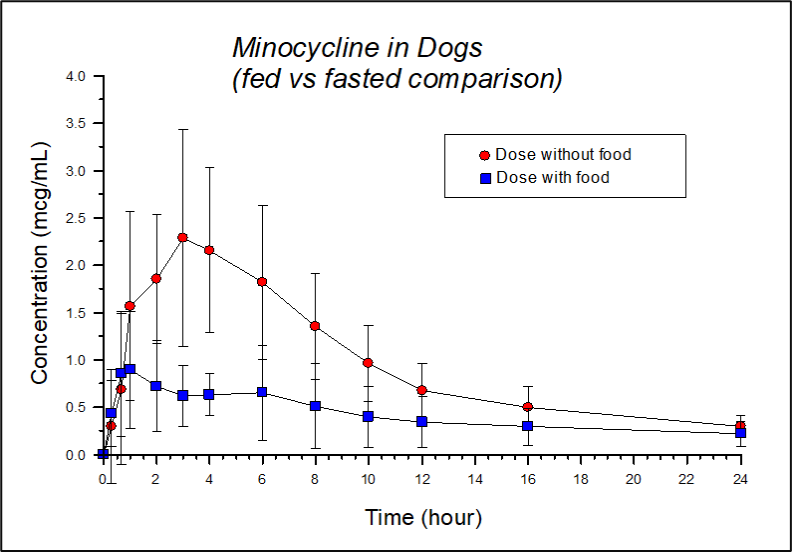
Minocycline oral absorption in dogs; comparison of fed vs. fasted. All dogs were administered the same dose in a crossover study (based upon data from Hnot et al. 2015) [24].

Although formulation comparisons are typically generated in fasted animals, we know that a prandial state can alter feline and canine oral drug absorption. Frequently, food will increase drug solubilization (reasons discussed below). However, as summarized by Watson (1979, 1986) [22,23], food can negatively influence the oral bioavailability of penicillins, cephalosporins, and tetracyclines. Hernot et al. (2015) showed that feeding significantly decreased the oral absorption of minocycline in dogs (Figure 1) [24].

In cats, the absorption of chloramphenicol from the palmitate ester pro-drug was significantly impaired if the cats fasted, while that of chloramphenicol tablets was not [25]. As discussed below, these two molecules exhibit similar solubility characteristics. These observations point to a range of factors that my influence postprandial differences in product bioavailability.

Examples of potential reasons for positive or negative food effects include [26,27]:

**Positive**: food-associated increase in gastric pH enhances drug solubility (e.g., weak acids); bile salts enhance drug solubility; food-induced increase in hepatic blood flow leads to saturated 1^st^ pass metabolism (e.g., propranolol); food-induced enhancement of lymphatic uptake, thereby bypassing first-pass hepatic metabolism.

**Negative**: food-associated increase in pH decreases drug solubility (e.g., weak bases); prolonged gastric residence time and drug instability in gastric fluids; increased fluid viscosity, which limits drug diffusion from dissolving tablet (retarding drug dissolution process); food-drug interaction (e.g., calcium and tetracycline); higher drug affinity for the bile micelle than for the intestinal unstirred water layer (micellar entrapment). The extent of the negative dissolution will depend upon the magnitude of the counteracting force imposed by the release of bile salts. The fats or fiber in a meal can lead to drug entrapment for certain compounds. Note that these effects are independent of pH-associated influences on in vivo drug solubilization.

When considering the decrease in free water and increase in fluid viscosity associated with a meal, humans tend to exhibit a decrease in tablet disintegration due to the decreased free water content. The meal-induced higher fluid viscosity (80 to 800-fold greater than that in the fasted human stomach) and the resulting decreased diffusivity can negatively impact in vivo dissolution [27,28]. Similarly, fluid viscosity can affect oral drug bioavailability in dogs. The nature of this viscosity-induced change varies as a function of the aqueous drug solubility and its site of absorption. Reppas et al. (1998) [28] observed that for highly soluble compounds absorbed primarily in the upper part of the small intestine, an increase in luminal viscosity would lead to a reduction both in peak concentrations and extent of absorption (drugs administered with guar gum to fasted dogs). In contrast, highly soluble compounds but absorbed throughout the GI tract are likely to exhibit a reduction in *C*_max_ without a decrease in AUC. However, the addition of guar gum had minimal impact on the AUC and *C*_max_ of poorly soluble compounds. We can anticipate similar effects in cats.

Regarding the other veterinary species, although fed/fasted studies may be published in horses [29] and swine [30], species such as cattle, horses, poultry, and swine typically have levels of residual material within their stomachs throughout the day. In nature, horses rarely exist in a fasted state [31]. Moreover, for food-producing species, much of the oral medications are administered either in drinking water or in medicated feed. Thus, for these species, oral drug bioavailability is generally determined by conditions prevailing during the fed state (note that equine oral bioavailability comparisons are accepted under fasted conditions by several non-US countries as evidenced by published equine papers) [32].

### Palatability

**Dogs and cats**: bad tasting or high dose drugs are a challenge to formulate into tablets that are freely accepted by dogs and cats. In addition to initial taste, mouth-feel and after-taste must also be considered. This has resulted in research efforts to support taste-masking technologies.

From an evolutionary perspective, it has been suggested that the canine ancestors may have relied not only upon animal prey but also upon plant materials when prey was scarce [33]. For this reason, dogs often consume foods containing either animal-derived or vegetable-derived flavors. While they prefer meat-based or complex flavor mixtures (including animal protein digests and hydrolysates, animal proteins, emulsified meats, amino acids, animal fats), they can also be attracted to sugars such as sucrose, glucose, fructose, and lactose (but not maltose) [34,35]. This behavioral observation is consistent with the neurophysiological response to these substances, the intensity of which is influenced by the presence of monovalent cations (e.g., Na^+^), divalent cations (e.g., Ca^2+^), and the amount of these ions relative that of the sugar [36].

In contrast, from an evolutionary perspective, cats remained dependent upon frequent meals of small prey. While both dogs and cats exhibit a carnivore pattern of taste preferences, cats further display a differential response pattern to certain animal acids (e.g., stimulated by L-lysine but inhibited by 2-tryptophan) [33]. They prefer flavors such as fish, liver, meat, sour/acidic flavors (pH range of 4.5–5.5), Brewer’s yeast, yeast extract, dairy (milk and cream), and amino acids [34]. They neither have an attraction nor aversion to sweet carbohydrates [37]. Based upon studies of taste-induced electrophysiological nerve activity, as with dogs, this behavior is consistent with a lack of neuronal stimulation [37].

Thombre, 2004 [34] noted that dosage form texture, shape, and size are considered to be more important to cats than dogs. This appears to be related to a cat’s tendency to nibble their food, while it is chewed and rapidly consumed by dogs. Cats prefer the ‘‘cheerio’’ or ‘‘star’’ to a ‘‘fish’’ shape, a finding that influences the manufacture of cat food more so than it does medicinal products.

Unlike the flavorants used in human medicine, many of those used for dogs and cats are lipophilic and may impose manufacturing and stability problems. This is particularly problematic when large amounts of the flavorants are needed to cover a very bitter drug substance [38]. Even when artificial flavors are used, many continue to pose lipophilicity concerns. In that regard, the greater the amount of lipophilic flavorants integrated into the tablet (particularly chewable formulations), the greater the risk of negatively impacting drug product dissolution (and stability).

Flavorants can either be natural or artificial. These terms are defined in 21 CFR 101, 22(a)(3).

**Horses**: the general preference tends toward sweet and salty flavors [39]. However, an in-depth analysis shows that taste preferences can be influenced by the animals' breed and sex. For example, pellets containing molasses were consumed more willingly by mares than stallions. Feeds with the addition of apples or carrots were the favorite treats of all tested breeds [40]. Although medications are frequently administered to horses as oral pastes or suspensions, tablet formulations have been approved. For example, in a study comparing firocoxib oral paste to an oral tablet, the peak exposure and time to peak exposure were slightly greater after the paste as compared to the tablets, although the extent of firocoxib oral bioavailability from these two formulations was comparable (NADA 141-458, https://www.equioxx.com/).

**Cattle, Pigs, and Poultry**: with regard to these other target animal species, the types of dosage forms used (see discussion above) limit the importance of palatability concerns to drug formulation.

## Prescribing practices

Due to human food safety concerns, it is not legally permissible to administer drugs in an extralabel manner to food-producing animal species such as cattle, swine, and poultry. In contrast, it is legal (and common) to prescribe drugs in an extralabel manner to dogs and cats [41]. Moreover, it is not unusual to find human generic drug formulations prescribed for administration to companion animals or for drugs approved for use in dogs to be prescribed for similar indications in cats. Therefore, it is important to appreciate potential interspecies differences in oral bioavailability and in vivo product performance.

Differences in gastric acid secretion can be particularly important in the solubilization and subsequent absorption of weak bases [42]. Since the dissolving free base can neutralize gastric acid, drug solubility may decrease during the gastric residence of a basic drug substance. This issue can be particularly problematic when extrapolating human drug solubility estimates because gastric acid secretion in dogs and cats is markedly lower than that of humans [43,44].

Examples of recognized in vivo differences in oral drug bioavailability are provided below.

## Examples of dog-cat differences in oral bioavailability

There are many factors influencing oral drug absorption across veterinary species, including drug solubility, GI transit time, absorptive surface area, active transporters, and metabolizing enzymes (enterocyte and the liver). While it is not always possible to distinguish the magnitude of which solubility versus permeability and presystemic metabolism contribute to these dog/cat bioavailability differences, some published examples show that drug solubility may have had an important role. Please note that the studies reported below have been conducted either using experimental formulations or drugs that may not have been FDA approved for use in the investigated species. The drugs discussed below were used solely to explore pharmacokinetic (PK) differences between dogs and cats and are not intended to characterize or imply therapeutic uses.

**Figure 2.**
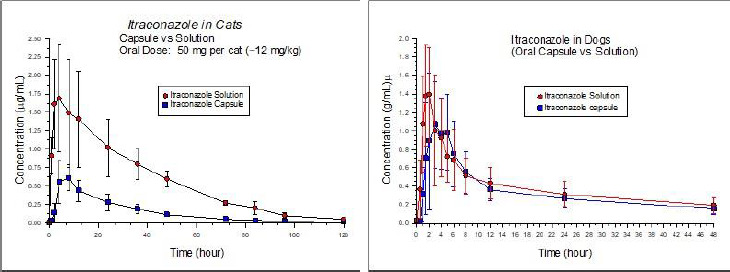
Oral absorption of itraconazole capsules vs. solution in cats (left) and dogs (right) From Hasbach et al. 2017, and Mawby, et al. 2018 [47,48].

- Chloramphenicol: chloramphenicol is a neutral molecule with an estimated water solubility of 2.5 mg/mL. Similar solubility characteristics are associated with the palmitate ester. However, the palmitate oral suspension is associated with markedly lower oral bioavailability as compared to chloramphenicol tablets. This is particularly evident when administered to fasted cats [25]. In contrast, equivalent palmitate and crystalline chloramphenicol doses produce comparable blood levels in the fasted dog [45]. The author suggests that, at least in part, the poor bioavailability of chloramphenicol from chloramphenicol palmitate in fasted cats might be due to reduced secretion of digestive enzymes in the fasting state and consequently impaired hydrolysis of the ester. Since the dog and the cat were both dosed with chloromycetin palmitate suspension by Parke Davis and Company, the observed species differences could not be attributed to potential differences in chloramphenicol palmitate polymorphic form.
- Cannabidiol (CBD): there is tremendous interest in the use of CBD in pets. However, poorly soluble CBD has lower oral bioavailability in cats than dogs. When administered to dogs in the form of oral chews (soft chew treat made with a glycerol/starch/fiber base), CBD had markedly higher oral absorption than CBD-infused fish oil capsules administered to cats. Both formulations were administered at a dose of 2 mg/kg. But the mean maximum concentration (*C*_max_) was 301 and 43 ng/mL in dogs and cats, respectively. The time to maximum plasma concentration (*T*_MAX_) values were 1.4 + 0.2 hr (dog) and 2.0 + 0.6 hr (cats). Species differences in terminal elimination half-life (*T*_½_) could not explain these outcomes as the *T*_½_ in dogs was 1 hr while that of cats was 1.5 hr. The corresponding area-under-the-curve (AUC) was 1297 and 164 ng hr/mL for dogs and cats, respectively [46]. The extent to which formulation versus species impacted this tremendous difference in absorption is unclear. However, since CBD itself is a highly lipophilic substance, at least in part, one may expect that differences may have been associated with a higher affinity of the CDB for the fish oil in the feline capsule (thereby preventing its solubilization within the GI fluids), than for the fluids in the feline GI tract. Other possibilities to consider include species differences in GI fluid composition, or, due to the use of an extruded formulation in dogs, could have induced a fed in the dog (thereby improving drug oral bioavailability).
- Itraconazole: this compound is notorious for its poor oral solubility. Oral absorption of this azole antifungal is highly affected by its solubility in the GI tract. In dogs, the oral absorption of oral itraconazole capsules and the oral solution (Sporanox, which is drug complexed in a hydroxylpropyl-β-cyclodextrin vehicle) are comparable (Figure 2). Relative bioavailability of the oral capsules vs. solution in dogs was 85 % [47]. In cats, there was a much greater difference between the oral bioavailability of the two dosage forms (Figure 2), likely reflecting species difference in GI pH, GI fluid volume (smaller in the cat) and potentially differences in endogenous surfactants (note that we currently lack information on bile salt composition in the cat). When the oral solution (Sporanox) was administered to cats, the bioavailability was approximately 5x higher than the oral capsule [48]. The pKa of itraconazole is 3.7 (a weak base).
- Fluoroquinolones: problems associated with the solubilization of ciprofloxacin in small fluid volumes is more pronounced in cats than in dogs. Accordingly, ciprofloxacin oral bioavailability in cats (10 mg/kg of ciprofloxacin powder in hard gelatin capsules) was only 22 % (%CV=50), rendering it unsuitable for treating most feline bacterial infections [49]. In dogs, dosing the oral solution (approximately 7.5 mg/kg) resulted in an oral bioavailability of about 78 % (%CV 26.4 %) and about 61 % (%CV=57.7 %) for an immediate release tablet formulation (dosed at approximately 20 mg/kg) [50].
- Selamectin: in contrast to the other two examples, selamectin has markedly higher oral bioavailability in cats than in dogs [51]. The oral formulation contained 24 mg selamectin/mL in sesame seed oil. A single dose of 24 mg/kg was given by oral gavage to cats or by a stomach tube in dogs. As compared to an intravenous dose, oral bioavailability was 109 % in cats but only 62 % in dogs. Nevertheless, *T*_max_ (7 hrs cat, 8 hrs dog) was comparable. Again, whether permeability/presystemic metabolism versus in vivo solubility was a primary cause of these differences is unclear. Selamectin is a P-gp substrate. While one may deduce the potential impact of species differences in intestinal P-gp, this appears unlikely since the tissue distribution of P-gp appears to be similar in dogs, humans, and cats [52].

## Summary of factors influencing drug solubility within the GI track the US major veterinary species

### Dog

A primary incentive for the amount of published research available on the canine GI tract relative to that available in other veterinary species has been the use of dogs as a preclinical species for the assessment of oral absorption, formulation strategies and the PK of medications being developed for human use [53]. However, because these preclinical studies are typically performed in fasted Beagle dogs of approximately 10 kg BW, there is a lack of information on how the canine GI fluid environment may vary as a function of age, breed, diet, and prandial state.

Under fasted conditions, the fluid volume of the Beagle stomach tends to be low. Using magnetic resonance imaging from 12 Beagle dogs weighing 9–12 kg (6 male and 6 female), the average gastric fluid volume was found to be 24.0 ± 4.2 mL [54]. That estimate is within the range of volumes (6 mL to 35 mL) used to examine the applicability of the human BCS system application for dogs [13].

The oral absorption of fluoroquinolone antimicrobials illustrates the impact of human-canine solubility differences and, at least in part, its contribution to species differences in oral bioavailability.

- Ciprofloxacin tablets are formulated for people and not for dogs. Although they can be administered extralabel manner, the oral absorption in dogs was shown to be lower and much more variable than that observed in people [55,56] (keep in mind that as discussed above, even lower oral bioavailability is expected to be associated with oral ciprofloxacin administration to cats). The authors suggest that one explanation for the low and variable oral absorption in dogs is their low gastric volume. The canine oral dose was administered with a 12 mL water flush. Although ciprofloxacin is classified as a highly soluble drug (based on BCS criteria), this assumes a fluid volume of 250 mL. Since its solubility is approximately 10 mg/mL, for even the largest sized human tablet (750 mg), the dose number (*D*_0_) for humans = 0.3. A *D*_0_ < 1.0 is considered highly soluble [57]. Clearly, a different situation exists in dogs when they consume the tablet with a water volume of 12 mL (the volume of the oral flush). With that small fluid volume, the calculated D_0_ for ciprofloxacin in dogs was 2.08, which would classify ciprofloxacin as a poorly soluble drug in dogs. In contrast, when ciprofloxacin was administered as an oral solution in the same study, it was dissolved in a much larger volume of water (37 mL). The D_0_ calculated for this volume was 0.68, rendering it highly soluble. Accordingly, a markedly higher fraction of administered dose was absorbed. The finding from that study is consistent with the work of Martinez et al., 2017 [50], where it was observed that unless the ciprofloxacin dose had been fully solubilized by the time it reaches the upper portion of the small intestine, it bypasses the canine absorption window, leading to poor oral bioavailability.
- A different situation exists for levofloxacin [58]. Levofloxacin tablets formulated for humans and not for dogs were nearly 100 % absorbed in dogs and did not exhibit the variability seen in the previous studies using ciprofloxacin. The higher and more predictable oral absorption of levofloxacin can be explained by its higher water solubility. With a reported water solubility of approximately 200 mg/mL, levofloxacin succeeds in meeting the *D*_0_ criteria of highly soluble in dogs.

There is little published information available on the composition of fluids in the canine GI tract in the fed state. However, there is evidence that in contrast to humans, canine food consumption does not produce an initial increase in stomach pH. This human-canine difference may be attributable to the lower basal peak acid secretion known to occur in dogs [53].

The USP GC<1236> provides the components and composition of biorelevant media describing the gastric and intestinal fluids of the fasted dog. Due to the variations in reported canine gastric pH values, the fasted gastric fluids are described for a pH range of 1.2 – 2.5 or 2.2 – 6.5. This is somewhat different from the pH range of 0.9 to 2.5 found using the Bravo^R^ capsule in dogs [59,60]. This range of variable results (particularly if it reflects within and between-dog variability) can affect our predictions of canine drug solubility.

The canine small intestines tends to exhibit a higher pH than that of humans. Accordingly, weak acids are typically more soluble in the canine vs human fasted intestinal fluids. For neutral molecules and weak bases with a pKa of less than 3, the difference is largely a function of the species-specific extent of bile micellization. Above pH 4, the human-canine differences in the solubility of weak acids appear to be associated with the extent to which solubilization occurs when the drug is in both its ionized and its unionized forms [61].

A comparison of human and canine bile acid composition was reviewed by Martinez et al., 2021 [7]. A fundamental difference between these two species is that while the ratio of human bile acid conjugation to glycine (primary) versus taurine (glycine/taurine) is 3, there is negligible conjugation to glycine in dogs. Nearly all of the canine bile acid conjugation is to taurine. Under fed conditions, intestinal bile salt and phospholipid content tend to be markedly higher in the dog (estimated in Labradors as 18mM and 19.4 mM, respectively) as compared to humans (11.8 mM and 4.31 mM, respectively). One might anticipate that these differences in intestinal components could lead to a greater ability to solubilize certain lipophilic drugs in dogs as compared to humans [9]. Furthermore, the intestinal buffering capacity (mM/ΔpH) of the fasted dog is slightly higher than that of fasted humans (13.8 versus 12 for dog and human, respectively) and the osmolality (mOsmol/kg) tends to be higher in humans as compared to dogs (181.6 versus 270 for dogs and humans, respectively) [61].

### Cats

Medication issues in cats have been reviewed by Papich (2006) [62] and Herve Lefebvre CVM Ph.D. DECVPT and Brice Reynolds DVM) [63]. These reviews note that less is known about the GI fluid composition of cats as compared to dogs. However, recent evidence shows that in addition to differences in GI fluid composition and gastric volume, the gastric pH of cats differs from that of dogs. For example, Tolbert et al. (2017) estimated the gastric pH of unanesthetized cats using Bravo capsules that were retained in the stomach, permitting measurements for a duration of 12 hrs [64]. When averaged over the 12-hr test period, the gastric pH of healthy cats was approximately 1.6. Although the cats were fed prior to capsule administration, there did not appear to be a change in gastric pH over the 12 hr period, even though cats fasted during this time.

Slightly different results were observed by Telles et al. (2021) when using a Bravo capsule to capture feline GI pH under fasted and fed conditions. In their study, the gastric and intestinal pH’s were higher under fasted versus fed conditions [65]. In addition, tremendous inter-animal variability was observed both for the gastric and intestinal pHs (Table 2).

Although not directly related to drug solubility, the following additional points need to be considered when formulating poorly soluble, slowly dissolving compounds for cats and may be of value to consider when developing in vitro dissolution procedures for feline solid oral dosage forms:

- The gastric emptying rate of 1.5 – 5 mm beads in the fed state is markedly longer than that in the fasted cat. The time to 90 % emptying (T90) was 0.74 hrs (1.5 mm bead) and 1.02 hrs (5 mm bead) in sedated fasted cats. In the fed state, the T90 of sedated cats was 6.65 hrs and 9.06 hrs for the 1.5 mm and 5.0 mm beads, respectively [66].
- The short intestinal transit time of 1.5 – 5 mm beads is unaffected by the prandial state and may be slightly longer in the cat than the dog [63].
- In cats, it is essential tablets are administered in a manner that prevents esophageal retention, which, in turn, can lead to esophageal stricture or esophagitis. This has been cited as an important problem for drugs formulated as acidic hydrochloride salts. The most often cited example is doxycycline hyclate (hydrochloride), which can cause severe esophageal lesions (Figure 4) [67,68].

When employing the Bravo capsule monitoring system to estimate GI transit times (TT) in fasted and fed cats (crossover study), Telles et al. (2021) [65] obtained the results given in Table 3.

Note that while the direction of differences in fed versus fasted states was comparable to that described by Chandler et al., 1997 [66], the observed transit times were somewhat different. This comparison is consistent with the influence of particle size and GI transit times.

- The maximum fluid volume (L) in the small intestine and colon of the cat is markedly less than that of the dog (Table 4) [69]. In addition, because of their inherent feeding habits (discussed above), cats have smaller stomachs and drink less water than dogs.

### Cattle

Forage and feeds mix with the bovine saliva that contains sodium, potassium, phosphate, bicarbonate, and urea. The resulting bolus moves from the mouth to the reticulorumen, which can hold approximately 5 gallons of material in the mature cow (liquid plus food). The latter compartment acts in a manner similar to that of a fermentation vat, containing microbes, carbohydrates, and a variety of volatile fatty acids (refer to USP GC <1236>). The abomasum is the “true stomach” of a ruminant. It is the compartment that is most similar to a stomach in a nonruminant, with a pH of approximately 3.5 to 4.0 due to hydrochloric acid secretion [70].

When considering drug solubility in ruminants, conditions in the rumen are typically the predominating factor. For example, in a study conducted on sulfamethazine oral boluses, ruminal conditions, including the slow transit of material through the rumen, allowed for formulations exhibiting markedly different in vitro dissolution profiles to produce nearly identical oral bioavailability [71].

Rumen pH is best measured at its lowest value (i.e., 2-4 hours after feeding a concentrate meal or 4-8 hours after offering a fresh total mixed ration). Rumen pH can be determined using a portable pH meter. The normal pH of grass-fed ruminants is 6-7. A pH value of 5.5-6 is seen in cattle on high-grain diets or pasture-fed cattle with early lactic acidosis [72].

As noted in USP GC <1236>, the normal pH of a healthy reticulo-rumen is in the range of 5.5–6.8. High grain diets typically result in a lower ruminal pH (~5.5), whereas high-forage diets result in a higher ruminal pH (~6.8). The USP GC <1236> also states that the pH of the abomasum (true stomach) is about 2–3 and that their intestinal pH is similar to that observed in monogastrics and humans. The pH at the pylorus is about 3.0 and increases to about 7.5 in the ileum (USP GC <1236>).

### Swine

An extensive study on the postmortem gastric and intestinal fluid contents of fasted Landrace pigs was published by Henze et al. (2020) [73]. This information will soon be incorporated into USP GC <1236>. Pigs were fasted for 24-hours prior to euthanasia. The authors compared the information obtained in their study to that published in dogs and humans (for references describing the GI fluid contents for humans, dogs, and minipigs to which the swine data were compared, please refer to the manuscript by Henze et al, [73]). They observed the following:

- pH
  - Gastric fluids: Similar to humans, porcine fasted state pH varies from 1.7 to 3.4, with a mean value of 2.2 ± 0.7 (median: 1.9). These values are slightly higher than the fasted pH reported in Yucatan minipigs (0.3–1.7).
  - Intestine fluids: Values ranged between 6.3 and 7.9, with a mean pH of 7.0 ± 0.5 (median: 7.0). The observed inter-subject variability was low, indicating a consistent and well buffered intestinal pH.
- Buffer capacity
  - Gastric fluids: Swine gastric buffer capacity is 6.1 ± 3.5 mmol * l^-1^ *△pH^-1^, which is lower than that reported for humans (14.3 ± 9.3 mmol* l^-1^ *△pH^-1^).
  - Intestinal fluids: Swine small intestine buffer capacity is 19.4 ± 2.9 mmol* l^-1^ *△pH^-1^, which is 3.4- fold higher than humans and 6.9- fold higher than dogs.
- Osmolality
  - Gastric fluids: The osmolality in the swine stomach is 99.33 ± 53.08 mOsm kg^-1^, which is lower than that of the human (mean of about 220 mOsm kg^-1^) but higher than that of dogs (74.9 mOsm kg^-1^)
  - Intestinal fluids: The osmolality in the swine small intestine is 387 ± 61 mOsm kg^-1^samples of landrace pigs was 2-fold higher than that reported for humans (about 197 mOsm kg^-1^) and that of dogs (69-207 mOsm kg^-1^).
- Bile: a major difference between the bile acids of humans versus pigs is that in humans, hyocoholic acid is only present in small amounts, whereas, in pigs, it is one of the key bile acids. In addition, while the most common bile acids in humans are cholic acid, chenodeoxycholic (CDC) and deoxycholic acid, in pigs, CDC and hyodeoxycholate acid are the major bile acids. 97.2 % of pig bile is conjugated with either glycine (69.3 %) or taurine (38.2 %). The total intestinal bile salt concentration ranged from 19.43 to 38.44 mM (median human estimate = 3.30 mM). Henze et al. (2020) [73] provide highly detailed supplemental information on the specific amounts of each bile acid contained in gastric and intestinal fluids.
  - Gastric fluids: Total bile acid content = 2.5 ± 1.7 mM
  - Intestinal fluids: Total bile acid content = 28.3 ± 9.6 mM
- Phospholipids: The most prevalent phospholipid in the swine GI tract is phosphatidylcholine, which is hydrolyzed to lyso-phosphatidylcholine in the small intestinal lumen. The swine phospholipid concentration is somewhat lower than that of humans.
  - Gastric fluids: Total phospholipids = 0.20 ± 0.18 mM
  - Intestinal fluids: Total phospholipids = 0.37 ± 0.20 mM
- Cholesterol: The high cholesterol intestinal content was interpreted as a function of enterohepatic recirculation of bile juices that contain bile salts and cholesterol. It is similar to that reported in humans.
  - Gastric fluids: Total phospholipids = 0.051 ± 0.060 mM
  - Intestinal fluids: Total phospholipids = 1.442 ± 0.772 mM.

Regarding GI transit time, this was estimated in pigs in a semi-fasted state. The pigs were administered either labelled solution, tablets or pellets and the transit time was monitored via gamma scintigraphy. Tablets and pellets were administered in a crossover study design. Furthermore, the pigs were administered a drink of 200 mL strawberry milkshake containing a colloidal Tc-99m colloidal solution. With this preparation, the time for 50 % gastric emptying of the liquid was approximately 1.4 hr, that of pellets was 2.2 hr, but the tablets movement from the stomach ranged between 5 and 6 hrs for 2 pigs and between 1.5 – 2 hr for another pig. The estimated total transit time for the various dosage forms was about 24-48 hours, except for one pig whose tablet total transit time was 72-96 hr [74].

### Horses

Horses are unique regarding oral absorption of medications. Although this topic was reviewed several years ago, the author’s observations remain relevant today [75].

Being herbivores, the equine GI tract is adjusted to a high carbohydrate and fiber diet. Regarding the time for gastric emptying of tablets in fasted horses, a range of values have been reported. For example, the *T*_MAX_ of acetaminophen plasma concentrations (which is used as a marker to reflect gastric emptying across a range of species, including humans) was estimated as 30 minutes in one study [76], but much between-animal variability was observed. In another study, corresponding fasted *T*_MAX_ values were estimated to range from 28-87 minutes [77]. Using nuclear scintigraphy, the time to 50 % versus 90 % gastric emptying was 30 and 138 minutes, respectively [78]. With respect to water, nearly complete movement from the stomach into the small intestine occurs within 10 minutes [79]. With regard to other sections of the GI tract, the small intestine comprises 30 % of the total digestive tract, but the passage of food is rapid at approximately 1 foot/minute going from duodenum to cecum. The hindgut is comprised of the cecum, large colon, small colon. Of primary importance are the fermentation activities occurring in the hindgut, where volatile fatty acids (acetic, propionic, and butyric acids) are produced, and water is reabsorbed [80]. Therefore, the “forgiving” solubilization conditions seen in cattle would not be applicable to the horse. The stomach of adult horses secretes approximately 1.5 liters of gastric juice per hour and has an acid output ranging from 4 to 60 mM hydrochloric acid per hour. The pH of gastric contents ranges from 1.5 to 7.0, depending on the region measured [81].

Bermingham et al. (2020) discussed some of the issues associated with bioavailability/bioequivalence studies in horses. They note that horses are typically fed a high fiber diet (e.g., hay) which increases gastric emptying time, affects gastric pH and potentially adsorbs administered drugs [31]. Specific values for gastric emptying time in fed horses depend upon what is being measured. Based upon information posted online by an equine nutritionist, the horse’s stomach is mostly empty by about six hours after being fed, with nearly all the larger fibrous particles passing within 12 hours [82]. Moreover, although it is possible to hold horses off-feed overnight to create a fasted state, intermittent feeding schedules that produce an empty stomach periodically may be one of the risk factors for equine gastric disease syndrome. Nevertheless, while some in vivo bioequivalence studies employ fasted horses (typically fasted for 8 – 12 hours), such studies do not replicate product oral bioavailability under actual field conditions (see the previous discussion on the prandial state). Care to avoid over-estimating oral drug bioavailability due to the tests being conducted in fasted horses rather than replicating prandial state under normal use conditions may be particularly important for antimicrobial agents where such over-estimations could potentially lead to ineffective treatments under field use conditions.

An additional problem is a tendency for some drugs to adsorb onto ingesta (hay, feed), preventing its aqueous solubilization and delaying its oral absorption until the ingesta reach the cecum, where it can be released upon digestion. This phenomenon can lead to the “double peaks” sometimes observed in the PK profile of drugs administered to horses. Baggot (1992) suggested that the double peak may reflect partial drug solubilization and absorption in the small intestine, while other portions require fermentative digestion prior to absorption in the colon or caecum [75].

Water-soluble drugs have been examined for bioavailability in the equine [83]. The study focused on cephalexin, marbofloxacin, metronidazole, and fluconazole (providing a wide range of physicochemical properties). The investigators observed that drug solubility had little influence on the drug’s oral absorption in horses. Rather, the authors noted that oral absorption was correlated more with the drugs’ lipophilicity (and ability to undergo transcellular absorption) than with its aqueous solubility. The exception was metronidazole which has a very low molecular weight and is likely absorbed by paracellular transport. Thus, the magnitude to which drug solubilization influences the observed fraction absorbed remains unresolved.

Another difference unique to the horse is that of its bile flow. Since horses lack a gall bladder, their bile flows continuously through the biliary tract into the duodenum. Food undergoing digestion leaving the stomach will quickly rise to a pH of 7.0 or slightly above [84].

### Poultry

Although the majority of poultry research has been conducted in chickens, for the most part, the information generated for the chicken GI tract can be applied to turkeys. A description of the chicken stomach, which consists of two chambers (proventriculus and gizzard), has been summarized by Zootecnica International [85]:

- **Proventriculus:** the site of acid secretion. Its thick walls are lined with gastric glands that secrete hydrochloric acid, pepsin, and mucus. This is the primary site of drug solubilization.
- **Gizzard**: the site of the physical digestion phase where most gastric proteolysis occurs. The muscles of the gizzard are arranged to allow for both a rotary and crushing action during contractions, thereby grinding the ingested feed. The gizzard epithelium is coated with a koilin layer which protects it from acid, proteolytic enzymes, and physical damage. The flow of feed particles into the small intestines is regulated by the pyloric sphincter, which restricts exit based upon particle size. The retention time of feed in the gizzard is about 30-60 minutes. In a review by Svihus (2011), he notes that the threshold size for being constrained from leaving the gizzard in chickens is between 0.5 and 1.5 mm [86]. He later notes that the gizzard content pH of broiler chickens varied between 1.9 and 4.5, with an average value of 3.5. However, due to the high calcium carbonate content in the diet, pH values for gizzard contents are commonly between 4 and 5 for layer hens, although a pH around 3.5 has also been reported for laying hens [87]. Note that the transfer of digested materials between the proventriculus and the gizzard can occur up to 4 times per minute.

Clearly, the GI environment can vary as a function of poultry breed and diet. Nevertheless, for estimation purposes, generalizations are often made based on broiler chickens. Gauthier (2002) summarized the pH and transit time (min) of poultry GI segments [88]. Table 5 represents the pH and mean transit time duration of all mash feed in different compartments of the broiler gut after 6-weeks of ad libitum feeding. Additional information on the enzymes within each section is based upon a symposium presentation by Rob Porter, 2012 [89].

## Impact on formulation strategies: how species GI fluid differences may impact formulation and how formulation can influence in vivo product performance

With these species-specific differences in mind, the following questions and answers provide perspectives on how formulations may need to be adjusted to optimize drug solubility (and therefore oral bioavailability) in veterinary medicine.

It should be noted that unlike the literature available for formulation optimization in human medicine, there is negligible corresponding information published for veterinary species. Therefore, this section reflects the perspectives of the authors based upon general published information and formulation principles. Where possible, we include citations to support our perspectives.

### How might poor drug solubility influence tablet formulation for dogs and cats?

Typically, formulation approaches used in human medicine [90] can be used to enhance the oral solubility of drugs administered to dogs and cats. However, the shorter GI tract and corresponding reduced time for drug absorption in dogs and cats introduce an additional challenge associated with formulation development. The other challenge is the smaller volume of fluid in the GI tract available for drug dissolution. This problem is even more pronounced in cats as compared to that of dogs.

Because solubilization must occur within a much shorter timeframe in dogs [7,91] and cats (see above), compounds exhibiting poor oral solubility may fail to attain the bioavailability needed to achieve therapeutic plasma drug concentrations. In these situations, it may be appropriate to administer the therapeutic moiety in a more readily soluble salt form [6] or as a prodrug. An example of this is the veterinary use of enrofloxacin versus ciprofloxacin [92]. Ciprofloxacin is known to exhibit inconsistent and poor oral bioavailability in dogs [55,56] and cats [49], while orally administered enrofloxacin is almost completely bioavailable in both species (https://bayer.cvpservice.com/product/basic/view/1040011).

### What are special formulation and manufacturing considerations needed when forming chewy, treat-like formulations?

Although we do not have information available on tablet hardness preferences for dogs, we do have some insights on that subject for cats.

When formulating feline chewable tablets, the generalized “ideal” hardness is in the range of 3–6 Kp. When hardness exceeds 6 Kp, tablet palatability tends to decrease. For example, while a tablet with a hardness of 6 Kp may be associated with a 95 % free choice acceptance, this value is reduced to 50 % free choice when formulated with a 12 Kp tablet hardness [93].

The texture is an issue for chewable medications and can make the tablet appealing or unappealing to dogs and cats. Although chewable tablets can be made by direct compression, wet granulation (using either water or alcohol), dry granulation (slugging or roller compaction), extrusion, or a forming machine [93], frequently, the “edible soft chew” is made using an extrusion or forming process. Both processes involve the mixing of the drug with tablet excipients (which include polymer, solvents such as polyethylene glycol (PEG) 30, glycerin, vegetable oil), binder, filler, stabilizer and flavorant) prior to the introduction of this mixture into an extruder or forming machine. The critical differences between these two processes pertain to the temperature and pressure at which the chews are produced, both being much higher for the extruder than the forming machine. These can be important considerations for heat-labile drugs or when using heat-sensitive solvents. However, both processes can be incorporated into the generation of tablets of varied sizes and shapes.

With regard to shape preferences, refer to the earlier discussion on tablet palatability.

### How might flavorants influence in vivo drug solubility?

Flavorants are available as commodity items from houses specializing in flavors for cats and dogs. Some potential flavorants are water-soluble (e.g., apple flavor or sugar or butter scotch flavor or lactose). However, others are poorly soluble, such as beef, liver, pork, chicken flavor, wheat germ, vegetable oil, or gelatin [94]. Species specific taste preferences have been discussed above (see the section on palatability).

The use of lipophilic flavorants can negatively impact the solubility of some compounds. In contrast, it could also potentially enhance the solubility of certain lipophilic compounds by inducing a semi-fed state and a release of bile salts. In that case, the positive or negative effect on in vivo dissolution will depend upon fed/fasted factors, as previously discussed.

### How might flavorants be incorporated into the formulation?

The manner in which the flavoring agents are integrated into the formulation depends upon the dosage form. For compressed tablets, if the flavorant comprises only a small percentage of the total tablet weight, no special manufacturing considerations are needed. However, the technique for taste masking may not simply be its addition as one of the tablet ingredients. For example:

- If the active agent is extremely bitter, taste-masking may need to be accomplished by drug encapsulation. This may involve additional manufacturing steps to coat the tablet with a polymer, adding complexity to the drug formulation.
- Alternatively, the addition of more flavorant may be needed to encourage consumption of chewable products manufactured by such processes as extrusion or forming machines. If the formulation is hard chewable, it can be made by direct compression, wet granulation (using either water or alcohol), or dry granulation (slugging or roller compaction). In that case, the flavoring agents can be applied using techniques similar to that used for human tablets.

### Are there excipients that may behave differently in dogs versus cats?

 Of particular concern is the effect that interspecies differences in GI pH and volume can have on the choice of disintegrant. Zhao and Augsburger [95] studied the effect of the pH on the disintegrant and concluded that an acidic medium significantly reduces the liquid uptake rate and capacity of sodium starch glycolate (Primojel) and croscarmellose sodium (Ac-Di-Sol) but not of crospovidone NF (Polyplasdone XL10). Tablets containing croscarmellose sodium were less affected by the acidic medium than were those formulated with sodium starch glycolate. Therefore, given the tendency towards a lower gastric pH in the cat than in the dog, disintegrants such as sodium starch glycolate and croscarmellose sodium may not function well when included in formulations intended for administration to cats. Furthermore, since many formulations are used both in dogs and cats, it may be preferable to use a disintegrant such as crospovidone.

It should also be noted that while excipients associated with enteric coating have been explored for use in dogs [96,97], the pH at which drug release occurs needs to be carefully evaluated to insure the ability to withstand fluctuations in canine gastric pH and the location of drug release does not compromise oral bioavailability (when considering the relatively rapid transit through the small intestine). Clearly, more work is needed on this topic, with a focus on the relationship between formulation selection and the drug molecule. With regard to cats, although there have been discussions of nutraceuticals such as enteric-coated S-adenosylmethionine (SAMe) in cats [98], there is a lack of PK-based evaluations of the impact of excipient selection on the in vivo performance of enteric-coated formulations. Therefore, more data are needed to ascertain whether the optimal choice of enteric coating for a given drug will be the same for dogs and cats.

### How can crystalline form and particle size influence the drug solubility and in vivo performance of medicated premixes?

Given the effect of crystalline form on drug solubility [99], it is important to consider retention time in the stomach, rumen, or gizzard. While a food-induced delay in gastric emptying and release of bile salts may reduce the impact of this effect in ruminants and swine, it may be a concern in poultry, particularly if the particle size is about 1 mm or less (thereby enabling it to leave the gizzard undissolved [86]). Should this occur, it could be associated with decreased therapeutic effects. An example of where this issue could be problematic is coccidiosis in poultry where the locally acting drug needs to be fully solubilized upon entering the duodenum [100]. Alternatively, given the prevailing conditions in the bovine rumen, in vivo drug solubilization may be more forgiving for formulations administered to cattle [101].

### What are issues to be considered with regard to species differences in critical formulation variables

With the possible exception of the dog, little work has been published on the influence of species-specific GI fluid environments on drug solubilization or on in vivo drug product performance. However, by combining what is known about the GI fluid conditions of each species, along with information obtained from studies supporting human drug development, we can anticipate some of the challenges that may be worthy of consideration.

For horses, ruminants, and pigs, medications are often administered to fed animals or in medicated feed. Therefore, drug adsorption onto the GI contacts may influence in vivo drug solubilization [102,103]. On the one hand, adsorption of drug molecules onto the surface of excipients or materials such as hay can reduce drug particle size and increase the surface area of the drug available to the dissolution medium. Conversely, if the forces of attraction are high, desorption may be retarded, and absorption compromised. Accordingly, the potential for drug adsorption may need to be considered when formulating dosage forms such as oral suspensions for horses or medicated articles for cattle and swine.

For drugs administered in medicated feeds to poultry, drug particle size and crystalline form will be essential considerations in product manufacture.

For dogs and cats, many of the critical formulation variables have been discussed above.

Additional points to consider are:

Ruminants: given the relatively high pH present in the rumen, there should be no problems in terms of the solubilization of acids. However, depending upon its pKa, weak bases may exist in an unionized form. For the latter, the presence of endogenous surfactants may be anticipated to enhance drug solubilization. In addition, the long residence time of material within the rumen may help promote the solubilization of the drug. Unless there is an excipient present that can affect drug permeability, intestinal transit time, or presystemic drug metabolism, once the dissolved medication moves into the small intestines, oral bioavailability will likely depend primarily on the drug’s PK properties.

Horses: in contrast to the other veterinary species, the horse has the capability to ferment food (with the corresponding presence of surfactants and volatile fatty acids) in the hindgut, which comprises more than 50 % of the GI tract capacity. Therefore, along with its acidic pH, one may expect that additional solubilization can occur with this region [104]. The question is then the pKa of the compound, the magnitude to which the drug has already been absorbed in the upper portion of the GI tract, and the extent to which drug absorption can occur within the lower GI tract of the horse.

Another key issue that has been explored in the horse is the efficiency of various types of formulations to prevent drug gastric exposure. For example, omeprazole can be degraded by gastric acids. In a study of 5 formulations, it was observed that while the enteric-coated formulations tended to have modestly higher *C*_max_ and AUC values as compared to buffered suspensions when administered fasted horses, these differences were not statistically (or clinically) significant [105].

## Closing comments

While species differences in GI fluid volume and composition can affect drug solubilization, this constitutes only one of the factors influencing species-specific drug absorption characteristics. Other variables include GI transit time (dictating the time available for drug solubilization/product dissolution), and permeability. Moreover, the absorption process itself can promote the presence of in vivo sink conditions by reducing the amount of dissolved drug within the gut, thereby promoting the dissolution of products containing drugs with borderline solubility. In addition, although not a topic for this review, enterocyte metabolizing enzymes, intestinal influx and efflux transporters, and the possible influence of microbial gut flora can impact the amount of free drug remaining within the GI tract (i.e., affecting the fraction absorbed). When considered in combination with species-appropriate dosage forms and palatability, these physiological characteristics influence the formulation and manufacturing considerations to be incorporated into the development of veterinary orally administered drug products.

Important information gaps continue to influence our ability to generate in vivo solubilization predictions. This includes:

1. Little to no information on breed differences in physiological surfactants, buffers, ionic composition, pH, and fluid volume. Studies to generate this information are greatly needed.
2. More information is needed on the influence of diet on GI fluid composition under fed conditions for nearly all veterinary species. This is particularly important for dogs and cats, where most oral bioavailability studies are conducted in the fasted state.
3. Since the human-pet interaction with dogs, cats and horses can cover their entire life span, more information is needed on changes in companion animal GI fluid composition with age.
4. There is also the need for information on intestinal fluid composition as a function of the specific segment of the GI tract.

While many of these issues will have less importance in species where oral drug products are administered in drinking water or in medicated feed, it will be an important consideration for dogs and cats. Furthermore, it would be helpful to have more information generated on the GI fluid composition in food-producing species and potential variability as a function of breed and diet. Such information could be of value in understanding how components of medicated feed or drug crystalline characteristics and particle size may influence in vivo drug absorption.

Within animal health, funding limitations challenge opportunities to explore these critical questions. Clearly, each of these points can influence drug and species-specific formulation optimization or improve the prediction of oral bioavailability difficulties that can occur when dogs and cats are administered oral formulations in a manner other than that indicated on the FDA-approved drug label. Hopefully, there will be a greater emphasis on filling these information gaps in the future.

## Figures and Tables

**Table 1.** Estimates of gastric fluid volumes of food-producing species and of the horse

Species	Gastric Fluid Volume, L	Gastric Residence Time, hr	Temperature, °C
Cattle	Rumen: 47	8	36.7-39.3
Swine	0.5	1	38.7-39.8
Horse	1.5	0.25	37.2-38.2
Chicken	0.01 (proventriculus + ventriculus)	2	40.6-43.0
Turkey	0.04 (proventriculus + ventriculus)	2	40.6-41.5

**Table 2.** GI pH estimates in fed and fasted cats using a Bravo capsule monitoring system [65]

	Fasted		Fed	
	Median	Range	Median	Range
Esophageal pH	7	3.5-7.8	4.5	2.9-6.4
Gastric pH	2.7	1.7-6.2	2	1.1-8.6
Small intestine	8.2	7.4-8.7	8.3	7.9-8.6
Large Intestinal pH	8.5	7.0-8.9	7.8	6.3-8.7

**Table 3.** GI TT estimates in fed and fasted cats using a Bravo capsule monitoring system [65]

	Fasted		Fed	
	Median	Range	Median	Range
Esophageal TT (min)	11	1-317	2	1-379
Gastric TT (min)	94	1-4101	1068	484-5521
Small Intestinal TT (min)	1350	929-2961	1534	442-2538
Total GI TT (min)	1733	1115-5741	2796	930-6590

**Table 4.** Small and large intestinal maximum fluid volumes of dogs and cats (BWs not provided) [69].

Species		Absolute, L	Relative% GI volume
Cat	Small intestine	0.11	14.6
	Colon	0.12	15.9
Dog	Small intestine	1.62	23.3
	Cecum	0.09	1.3
	Colon	0.91	13.1

**Table 5.** pH, transit time, and enzymes associated with the various portions of the broiler GI tract [88,89]

GI Segment	Transit time, min	pH	Enzymes
Mouth	-	7-7.5	Amylase
Crop	50	5.5	None - mucous secretion
Proventriculous and Gizzard	90	2.5-3.5	Pepsin, lipase
Duodenum	5-8	5-6	Amylase, trypsin, collagenase, bile, lipase
Jejunum	20-30	6.5-7	Maltase, lactase, peptidases
Ileum	50-70	7-7.5	-
